# Brain activation during disorder-related script-driven imagery in panic disorder: a pilot study

**DOI:** 10.1038/s41598-019-38990-0

**Published:** 2019-02-20

**Authors:** Alexander Burkhardt, Christine Buff, Leonie Brinkmann, Katharina Feldker, Bettina Gathmann, David Hofmann, Thomas Straube

**Affiliations:** Institute of Medical Psychology and Systems Neuroscience, University of Muenster, Von-Esmarch-Str. 52, 48149 Muenster, Germany

## Abstract

Despite considerable effort, the neural correlates of altered threat-related processing in panic disorder (PD) remain inconclusive. Mental imagery of disorder-specific situations proved to be a powerful tool to investigate dysfunctional threat processing in anxiety disorders. The current functional magnetic resonance imaging (fMRI) study aimed at investigating brain activation in PD patients during disorder-related script-driven imagery. Seventeen PD patients and seventeen healthy controls (HC) were exposed to newly developed disorder-related and neutral narrative scripts while brain activation was measured with fMRI. Participants were encouraged to imagine the narrative scripts as vividly as possible and they rated their script-induced emotional states after the scanning session. PD patients rated disorder-related scripts as more arousing, unpleasant and anxiety-inducing as compared to HC. Patients relative to HC showed elevated activity in the right amygdala and the brainstem as well as decreased activity in the rostral anterior cingulate cortex, and the medial and lateral prefrontal cortex to disorder-related vs. neutral scripts. The results suggest altered amygdala/ brainstem and prefrontal cortex engagement and point towards the recruitment of brain networks with opposed activation patterns in PD patients during script-driven imagery.

## Introduction

Panic disorder (PD) is a frequent disorder with up to 3.1% 12-month-prevalence^1^ that causes substantial impairment to the individual. Patients suffering from PD are consistently concerned about the occurrence of a panic attack^2^ which is characterised by a sudden outburst of overwhelming fear triggering severe physical reactions^3^. According to cognitive models and behavioural studies, the occurrence of panic attacks is mainly driven by altered threat processing in PD patients^4–6^. For example, they appear more vigilant to internal bodily sensations^7^, and tend to misinterpret them in a threatening manner^8–12^. This misinterpretation bias appears to facilitate, among other factors, the initiation of panic attacks and may consequently result in the preservation of high levels of anxiety as well as the maintenance of the disorder^5,13^.

Neurobiological models suggest that altered threat processing in PD is related to dysfunctions within a brain network comprising the amygdala, the insula, the prefrontal cortex (PFC), the hippocampus, and brainstem sites, such as the periaqueductal grey (PAG) region and locus coeruleus^14–16^. A key role in altered threat processing in PD has been attributed to the amygdala^15,17^, which is assumed to reflect increased vigilance towards threat^14,15^. However, the precise function in the pathophysiology of PD remains to be resolved, since findings remain equivocal and altered threat-related amygdala activity has not been consistently reported^16,18,19^. A recent meta-analytical work in the functional neuroanatomy of panic disorder condensed findings, revealing abnormal activity predominantly in an extended fear network comprising brainstem, anterior and midcingulate cortex (ACC and MCC), insula, and lateral as well as medial prefrontal cortex, while aberrant amygdala activation seems to more strongly hinge on particular stimuli and experimental paradigms, sample characteristics, as well as on limited neuroimaging methods^16^.

Aside the amygdala, altered threat processing in PD patients appears related to aberrant medial PFC and ACC recruitment, with studies showing both increased and decreased activation in response to threat^20–24^. Aberrant activity in the PFC and the ACC was considered to reflect a deficiency in top-down control of threat responses in PD patients^14^. Furthermore, emerging evidence points towards threat-related functional perturbations within the brainstem and the insula in PD patients^15,25–28^. For example, a recent study revealed an association between higher subjective levels of anxiety and brainstem activity as well as increased activity of the insula, interpreted as a token of increased interoceptive processing in PD patients^25^.

Taken together, the neural correlates of altered threat processing in PD remain inconclusive, but ravel around functional perturbations within the amygdala, the PFC, the ACC, the brainstem and the insula. Results and interpretation of functional imaging studies might depend on varying experimental and analytical methods. Further investigations using controlled symptom provocation designs are warranted to gain a comprehensive understanding of the neurobiological underpinnings of altered threat processing in PD patients.

A promising tool to explore threat processing mechanisms in patients proved to be the method of script-driven imagery^29–34^. Participants typically listen to an emotionally laden narrative script and imagine the active role of the protagonist. It has been shown that the emotional and bodily reactions, resulting from threat imagination, are similar to the physiological arousal when facing *in vivo* threat^34,35^. The method has already been implemented in psychophysiological PD studies and revealed that the imagination of disorder related narrative scripts elicited increase startle, heart rate, and blood pressure responses in PD patients^10,36,37^.

Besides psychophysiological responses, recent neuroimaging studies suggest that script-driven imagery involves the recruitment of a fear-related brain circuitry including increased activation of the amygdala, insula, cingulate cortex, and the prefrontal cortex (PFC), as well as reduced activation of the ventromedial PFC (vmPFC) in anxiety patients^19,24,38–42^. Given the suggested role of these brain regions as described earlier, script-driven imagery might elicit increased vigilance, increased interoceptive processing, and deficient control of responses to threat in PD patients. We therefore hypothesise that aberrant activation particular for mental threat imagery is to be expected in these brain regions during the present experimental procedure.

Remarkably, only one fMRI study has used this method in a pilot study with a highly restricted PD sample (*n* = 6) until today and found threat-related increased activation in cingulate cortex and lateral PFC^24^. Thus, relevant brain imaging studies are lacking that use script-driven imagery as a powerful tool to better understand threat-related brain activation patterns in PD patients during a controlled but symptom provoking experimental situation.

To pursue this auspicious avenue in research, the aim of the present fMRI study was to explore neural correlates of threat processing in a suited sample of PD patients by means of script-driven imagery based on a newly developed set of controlled disorder-related and neutral scenarios. To this end, we presented the disorder-related and neutral narrative scripts to PD patients and healthy controls (HC) and investigated brain activation in several brain regions, suggested to be relevant for threat processing in PD patients, comprising the PFC, cingulate cortex, amygdala, brainstem, and the insula^43^. We would like to mention that, given the methodological limits of a small sample size, medication and comorbidities of patients as well as the lack of high resolution imaging to characterise brain stem responses, the present study should be looked upon as a pilot study.

## Results

### Rating data

**Figure 1:**
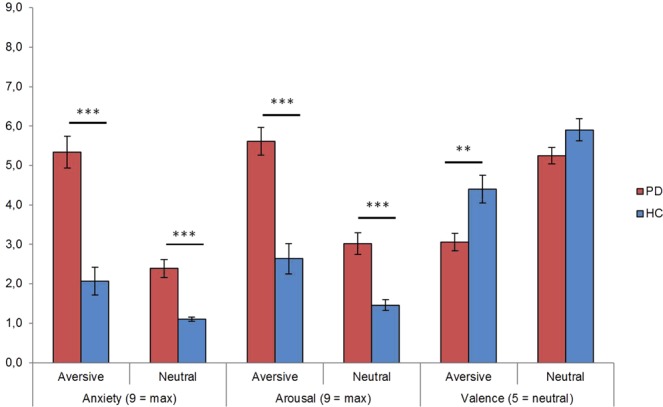
Mean ratings for disorder-related and neutral scripts on the dimensions of valence, arousal, and anxiety. There were significant interaction effects concerning all ratings: valence, arousal, and anxiety. Patients suffering from panic disorder (PD) compared to healthy controls (HC) rated disorder-related relative to neutral scripts as more anxiety-inducing, more arousing, and more unpleasant. ***p < 0.001, **p < 0.01.

Mean ratings are provided in Table 1. Analysis of valence, arousal and anxiety rating data revealed significant main effects of group (valence: *F*_[1,32]_ = 7.84, *p = *0.009; *arousal*: *F*_[1,32]_ = 35.83, *p* < 0.001; *anxiety*: *F*_[1,32]_ = 40.70, *p* < 0.001) and stimulus (valence: *F*_[1,32]_ = 185.90, *p* < 0.001; *arousal*: *F*_[1,32]_ = 185.90, *p* < 0.001; *anxiety*: *F*_[1,32]_ = 87.25, *p* < 0.001). Furthermore, there were significant *stimulus* by *group* interaction effects for *anxiety* (F_[1,32]_ = 22.41, p < 0.001), *arousal* (F_[1,32]_ = 12.49, p = 0.001) and *valence* (F_[1,32]_ = 6.34, p = 0.017) (see Fig. 1). Disorder-related scripts were generally rated as more arousing, more unpleasant, and more anxiety-inducing than neutral scripts. Patients rated all stimuli as more arousing and anxiety-inducing compared to HC, but neutral stimuli not as more unpleasant (t_[32]_ = −1.92, p = 0.064). Resolving the interaction effects for valence, arousal, and anxiety ratings revealed that disorder-related scripts were rated as more arousing (t_[32]_ = 5.70, p < 0.001), more unpleasant (t_[32]_ = −3.22 p = 0.003), and more anxiety-inducing (t_[32]_ = 6.11 p < 0.001) by PD patients compared to HC. Analysis of the *ability to imagine* each script yielded no significant effects (all p > 0.069).

**Table 1:** Mean rating data for disorder-related and neutral scripts on the dimensions of anxiety, arousal, valence, and ability to imagine the scripts.

Group	Script valence	Anxiety M (SD)	Arousal M (SD)	Valence M (SD)	Ability to imagine the scripts M (SD)
PD patients	Disorder-related	5.34 (1.66)	5.61 (1.45)	3.06 (0.90)	82.15 (6.93)
	Neutral	2.39 (0.96)	3.02 (1.14)	5.25 (0.84)	79.95 (6.88)
HC	Disorder-related	2.07 (1.45)	2.64 (1.59)	4.40 (1.46)	76.68 (9.80)
	Neutral	1.11 (0.21)	1.46 (0.55)	5.91 (1.14)	80.04 (9.46)
Overall	Disorder-related	3.70 (2.26)	4.12 (2.13)	3.73 (1.38)	79.42 (8.81)
	Neutral	1.75 (0.94)	2.24 (1.19)	5,58 (1.04)	79.99 (8.15)
PD patients	Across valence	3.86 (2.00)	4.31 (1.84)	4.15 (1.40)	78.36 (9.64)
HC	Across valence	1.59 (1.13)	2.05 (1.32)	5.15 (1.50)	81.05 (6.89)

Note. PD, panic disorder; HC, healthy controls; M, mean; SD, standard deviation.

### FMRI data

**Figure 2:**
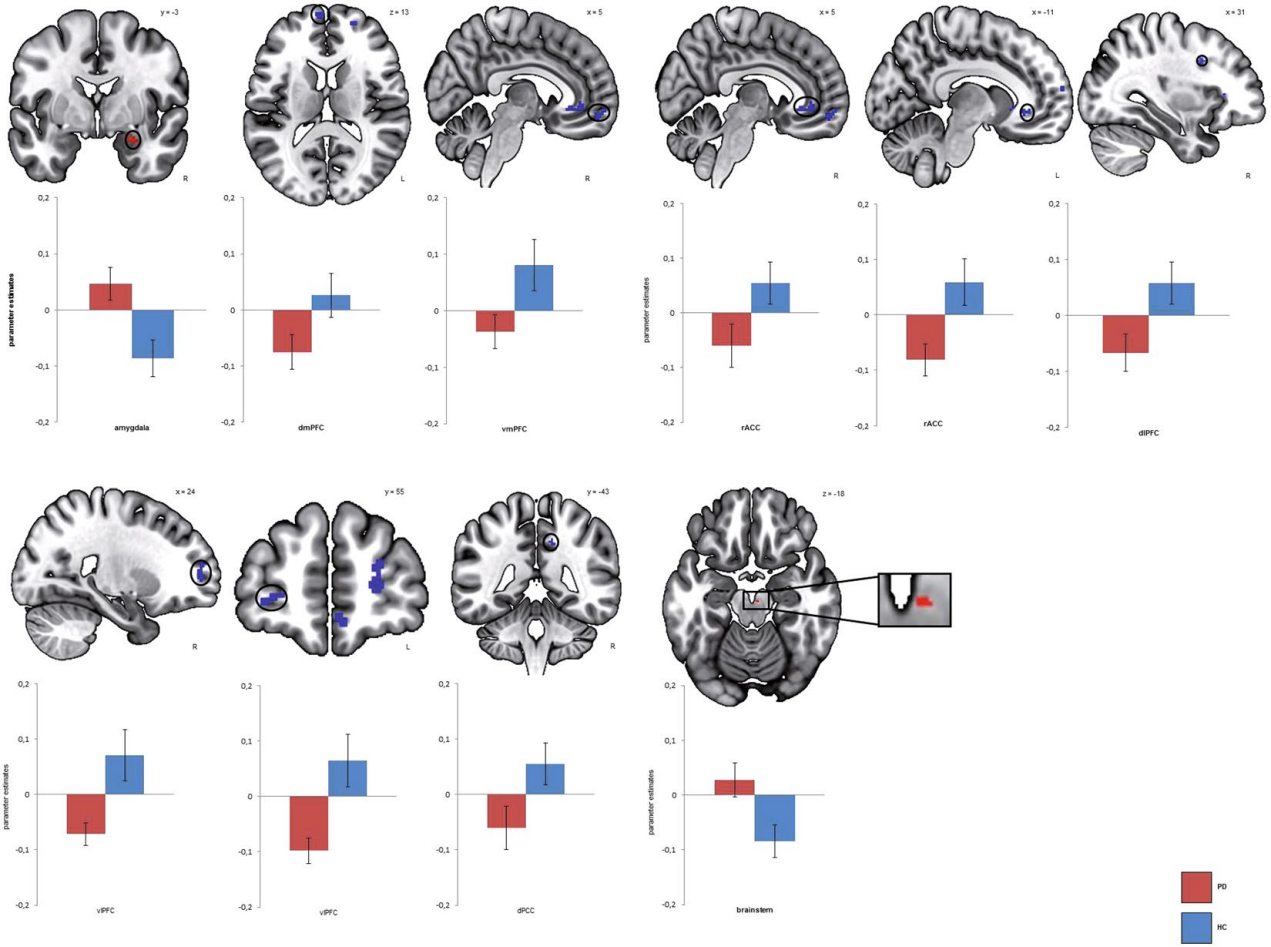
Differential brain activation during imagery exposure, disorder-related > neutral scripts. Panic disorder patients (PD) relative to healthy controls (HC) responded to disorder-related vs. neutral scripts with increased activity in right amygdala and brainstem, and decreased activation in right ventromedial prefrontal cortex (vmPFC), right and left ventrolateral prefrontal cortex (vlPFC), right dorsolateral prefrontal cortex (dlPFC), right and left rostral anterior cingulate cortex (rACC) and right dorsal posterior cingulate cortex (dPCC). Graphs indicate contrasts of parameter estimates (mean ± standard error) per group.

In response to disorder-related vs. neutral scripts, PD patients showed greater activation in the right amygdala (peak voxel Talairach coordinates: *x* = 24, *y* = −3, *z* = 19; max. t-value: *t*_[33]_ = 3.37, p < 0.05 corrected; cluster size 144 mm^3^) compared to HC. Furthermore, patients showed decreased activation in several prefrontal regions for the same contrast: left ventrolateral PFC (vlPFC) (peak voxel Talairach coordinates: *x* = −27, *y* = 40, *z* = 3; max. t-value: *t*_[33]_ = −4.45, p < 0.05 corrected; cluster size 1184 mm^2^), right vlPFC (peak voxel Talairach coordinates: *x* = 22,y = 25, *z* = 5; max. t-value: *t*_[33]_ = −3.69, p < 0.05 corrected; cluster size 448 mm^3^), right ventromedial PFC (vmPFC) (peak voxel Talairach coordinates: *x* = 14, *y* = 61, *z* = −4; max. t-value: *t*_[33]_ = −4.13, p < 0.05 corrected; cluster size 408 mm^3^) as well as left dorsomedial PFC (dmPFC) (peak voxel Talairach coordinates: *x* = −4, *y* = 62, *z* = 11; max. t-value: *t*_[33]_ = −3.55, p < 0.05 corrected; cluster size 208 mm^3^) and right dorsolateral PFC (dlPFC) (peak voxel Talairach coordinates: *x* = 31, *y* = 10, *z* = 33; max. t-value: *t*_[33]_ = −3.28, p < 0.05 corrected; cluster size 168 mm^3^). Additionally, decreased activation was found in several regions of the cingulate cortex: in left rostral ACC (rACC) (peak voxel Talairach coordinates: *x* = −11, *y* = 32, *z* = −7; max. t-value: *t*_[33]_ = −3.03, p < 0.05 corrected; cluster size 168 mm^3^), in right rACC (peak voxel: *x* = 7, *y* = 31, *z* = −6; max. t-value: *t*_[33]_ = −3.47, p < 0.05 corrected; cluster size 592 mm^3^) as well as in dorsal posterior cingulate cortex (dPCC) (peak voxel Talairach coordinates: *x* = 14, *y* = −44, *z* = 38; max. t-value: *t*_[33]_ = −3.33, p < 0.05 corrected; cluster size 72 mm^3^). Further inspection of brainstem ROI showed marginally significant hyperactivation in right brainstem to disorder-related vs. neutral scripts for PD patients compared to HC (peak voxel Talairach coordinates x = 3, y = −20, z = −18, max. t-value: *t*_[33]_ = 3.00, p < 0.005 uncorrected; cluster size 16 mm^3^) (Fig. 2). All significant activations in ROIs during imagination of disorder-related vs. neutral scripts are depicted in Table 2.

**Table 2:** Region of interest analysis: Significant activations during imagination of disorder-related vs. neutral scripts.

Region	Lateralization	Talairach coordinates of peak voxel			mm^3^	Maximum t-value
		x	y	z		
**PD > HC**						
Amygdala	R	24	−3	19	144	3.37
Brainstem*	R	3	−20	−18	16	3.00
**HC > PD**						
vmPFC	R	14	61	−4	408	−4.13
vlPFC	L	−27	40	3	1184	−4.45
vlPFC	R	22	55	5	448	−3.69
dmPFC	L	−4	62	11	208	−3.55
dlPFC	R	31	10	33	168	−3.28
dPCC	R	14	−44	38	72	−3.33
rACC	L	−11	32	−7	168	−3.03
rACC	R	7	31	−6	592	−3.47

Note. PD, panic disorder; HC, healthy controls; vmPFC, ventromedial prefrontal cortex; vlPFC, ventrolateral prefrontal cortex; dmPFC, dorsomedial prefrontal cortex; dlPFC, dorsolateral prefrontal cortex; dPCC, posterior cingulate cortex; rACC, rostral anterior cingulate cortex; L, left; R, right; (x, y, z), Talairach coordinates of maximally activated voxel. If not stated otherwise activations listed were calculated at p < 0.05 (corrected), *p < 0.005 (uncorrected).

## Discussion

The present study aimed at unveiling neural correlates of altered threat processing in PD patients by means of script-driven imagery. The disorder-related narrative scripts were rated as more arousing, unpleasant, and anxiety-inducing by PD patients as compared to HC. Moreover, PD patients relative to HC showed enhanced amygdala and brainstem activity and decreased activity in dmPFC, dlPFC, vlPFC, vmPFC/rACC and dPCC during imagination of disorder-related vs. neutral narrative scripts.

To the best of our knowledge, the present study is the first to reveal enhanced amygdala activity in PD patients during the imagination of disorder-related scripts. Absent differential amygdala activity in PD patients in a former script-driven imagery study might have been due to signal dropouts and very small sample size (n = 6)^24^. In line with our findings, increased amygdala activity was found in PD patients during the processing of fearful facial expressions^44,45^, symptom provocation^46,47^, an emotional Stroop task^48^, and processing of disorder-related pictures^25^. Nevertheless, other studies did not reveal threat-related differential amygdala activity in PD patients (for review see^18^). The amygdala has been suggested to play a role in attentional-vigilance aspects of detecting and processing emotional stimuli in normal and pathological forms of anxiety^49–52^, and aberrant activity has been associated with hyperarousal towards threat in anxiety disorders^53–55^. The amygdala also proved to be hyperresponsive to subtle environmental cues, triggering intense physiological threat-related responses in anxiety disorders as well as in healthy subjects^41,56^. We suggest that designs, in which subjects are strongly trying to control or label their emotions, lead to other brain activation patterns with even reduced amygdala activation (see also^57–60^). We infer from these findings that PD patients experienced ongoing, exaggerated vigilance and arousal during the imagination of disorder-related scripts. Furthermore, we would like to note that we do not suggest that the amygdala is necessary for the experience of panic attacks, since such attacks are also possible without amygdala involvement^61,62^. However, the amygdala might reduce the threshold for elicitation of such attacks due to their functions described above.

Apart from increased amygdala activation, we found brainstem hyperactivation in PD patients compared to HC during the imagination of disorder-related scripts. However, these findings have to be interpreted tentatively as brainstem hyperactivation only reached marginal significance (see Table 3). Increased activity in brainstem sites is in accordance with a recent study^25^, showing a link between increased activity in the brainstem and the subjective degree of anxiety evoked by threatening pictures in PD patients. Generally, the brainstem appears to be relevant for autonomic body functions, such as cardio-respiratory control, and seems to be associated with different levels of anxiety and anticipation of threat^63,64^. In PD, brainstem sites, such as the PAG, proved to be hyperactive during a face‐word interference task^21^ and in the safe condition of a fear conditioning paradigm^65^. In a “carbon dioxide hypersensitivity theory of panic”, it was posited that PD patients hold an altered suffocation alarm monitor, leading to a pathological carbon dioxide hypersensitivity that provides the basis for panic attacks^66^. This hypothesis is supported by recent findings, showing that increased brainstem activation and its correlation with the subjective fear of respiratory symptoms might present a neurobiological basis of such a monitoring system^67^. Supporting this, increased activity in the rostral raphe nuclei was demonstrated in response to hypercapnia^68^. In this context, increased brainstem activity in PD patients in the present study may hint at heightened experience of subjective fear of bodily symptoms during threat imagination in PD patients. However, as mentioned before, these conclusions have to be drawn cautiously due to marginal significance of brainstem activation. Questionnaires on the feelings of suffocation, CO2-monitoring as well as a more nuanced analysis of brainstem subnuclei in future investigations may aid in understanding on the role of altered brainstem activity patterns during threat imagination in PD patients.

**Table 3:** Demographics and questionnaire measures.

	Range	PD M (SD)	HC M (SD)	*t*-value	*p*-value
Age in years	19–35	24.12 (4.55)	24.35 (3.37)	0.17	0.865
Years of education	10–13	12.47 (0.72)	12.59 (0.80)	0.45	0.654
HAM-A	0–56	13.29 (7.76)	0.24 (0.56)	6.92	<0.001
STAI-Trait	20–80	46.53 (8.02)	30.24 (6.97)	6.33	<0.001
BDI-II	0–63	14.29 (6.86)	1.59 (2.48)	7.18	<0.001
ASI	0–64	49.00 (12.76)	13.35 (11.01)	8.72	<0.001
PAS	0–52	21.82 (6.31)	0.18 (0.53)	14.10	<0.001

Note. PD, panic disorder patients; HC, healthy controls; M, mean; SD, standard deviation; HAM-A, Hamilton Anxiety Rating-Scale^116^: <17: mild anxiety symptoms; STAI-T, State-Trait Anxiety Inventory^117^: >40: pathologically increased level of anxiety; BDI-II, Beck Depression Inventory^118^: >13–19: mild depressive. symptoms; ASI, Anxiety Sensitivity Index^119^: 0–64, (no standard value); PAS, Panic and Agoraphobia Scale^103^: 19–28: moderately severe PD.

PD patients were also marked by reduced vmPFC/rACC activity during disorder-related imagery. Impairment of vmPFC/rACC recruitment appears to relate to dysfunctional modulation of fear responses across anxiety disorders^25,41,43,69–71^ and has been observed in studies investigating implicit emotion regulation in HC^63,71,72^. In fact, the present finding of reduced threat-related vmPFC/rACC activity in PD patients constitutes a common finding across anxiety disorders, since ample evidence revealed reduced vmPFC activity during threat exposure in patient samples^43^. Yet, the former fMRI pilot study on threat-related script-driven imagery in PD patients revealed elevated, instead of reduced ACC activity in PD patients^24^, rendering further investigations necessary. All in all, we assume that reduced activity in the vmPFC/rACC during disorder-related imagination may indicate deficient regulation and modulation of emotional reactivity in PD patients. Out of interest, we conducted a within-group comparison of fMRI data exploring differences between PD patients with and without agoraphobia. This analysis interestingly revealed more pronounced decreased activity of medial prefrontal cortex and rostral anterior cingulate cortex in PD patients with agoraphobia compared to patients without agoraphobia. Given the depicted functions of these brain regions, agoraphobic patients seem even to a higher extend exhibit impaired implicit and explicit emotion suppression and increased focusing when imagining potential threat. This is in line with findings showing that the comorbidity of agoraphobia in patients with panic disorder causes a more severe disturbance compared to PD patients without agoraphobia^73,74^.

Another finding of the present study relates to reduced dmPFC activity in PD patients when imagining disorder-related scripts. The dmPFC seems to be involved in the explicit evaluation and appraisal of threat^70,75–77^. In PD patients, hypoactive dmPFC activity was found during deliberate reappraisal of negative emotions^78^ and during emotional conflict paradigms^21^. The findings hint at reduced resources of dmPFC recruitment during demanding emotional processing and reappraisal of negative emotions in PD patients. This may eventually lead to high anxiety levels in PD patients. Taken together, we assume that decreased vmPFC/rACC, together with decreased dmPFC activity during disorder-related script-driven imagery, may reflect deficient regulation of emotional reactivity as well as a dysfunctional appraisal and evaluation of threat in PD patients.

Moreover, disorder-related imagery induced decreased activity in the vlPFC as well as in the dlPFC in PD patients relative to HC. Both dlPFC and vlPFC constitute part of a frontoparietal executive network that was shown to mediate intentional, explicit evaluation and regulation of stimuli^57,71^. Correspondingly, the vlPFC was related to the suppression of emotional processing of negative pictures and the resulting anxiety responses, often with concurrent aberrant amygdala activity in anxiety disorders as well as in healthy subjects^20,79–82^. With regard to PD, findings of a study using panic-related words in a go/no-go task showed decreased activity in the vlPFC^20^ that was interpreted to indicate deficient emotion suppression functioning. Reduced vlPFC activation during script-driven imagery may therefore reflect dysfunctional, deliberate modulation of emotional reactivity in PD patients. In a similar vein, the dlPFC is assumed to regulate emotional responding to threat and to prevent excessive emotional expressions^57,71^. In PD patients, an emotion regulation study showed that patients responded with decreased activation of the dlPFC during the reappraisal of threat, which may hint at emotion regulation deficits^83^. In that respect, the present finding of reduced dlPFC activity during imagination of disorder-related scripts in patients is in accordance with previous findings and hints towards altered modulation of emotional responding in patients. It remains to note that the former fMRI pilot study on script-driven imagery in PD revealed the opposite pattern with elevated instead of reduced dlPFC activity in patients^24^, warranting forthcoming investigations. Altogether, decreased lateral PFC activation in PD might indicate that PD patients may have failed to intentionally dampen their excessive emotional response to the imagination of disorder-related stimuli.

Decreased activity in response to disorder-related scripts was furthermore found in the dPCC in PD patients relative to HC. The PCC has been suggested to play an important role in arousal and directed attention processes^84^. While increased activity of the PCC was observed in resting or passive task states^85^, directed attention on external stimuli seems to be associated with decreased PCC activity in HC^86,87^. Particularly the dorsal part of the PCC appears to be involved in detecting external stimuli that might demand a behavioural response to change situational conditions^86^. Correspondingly, decreased activity of the dPCC was observed in HC in tasks when a narrow focus on external stimuli was needed^87^. Since PD patients show a decrease in dPCC activity during disorder-related imagery, we thus speculate that patients fostered more focused attention to disorder-related scripts as compared to HC.

Interestingly, several regions found to be hypoactivated in the present study (i.e., mPFC, rACC, and dPCC) closely correspond to the so-called default mode network (DMN)^88^. The DMN is a neural network system that mediates levels of cognitive activity, showing lower activity during specific goal-directed behaviours^89^. It has been suggested that, as a result of direct competition with other systems, focused attention on external stimuli is inclined to attenuate activity in the DMN^85^. These findings lend preliminary support for the interpretation that the observed brain response pattern of the present investigation may reflect a stronger attentional focus on disorder-related scripts in patients relative to HC.

Script-driven imagery has predominantly been used in anxiety patients in order to research fear-related threat responses^10,24,90–95^. Therefore, it proves to be difficult to ascribe certain neural activation patterns to the paradigm itself. Activation patterns elicited by the paradigm may overlap disorder-specific activation patterns and similarities cannot be completely ruled out. Thus, results have to be carefully interpreted when it comes to draft a model of neural correlates specifically being involved during script-driven imagery. As mentioned before, script-driven imagery hitherto involved aberrant brain activation in the amygdala, insula, cingulate cortex, and the prefrontal cortex (PFC), as well as reduced activation of the ventromedial PFC (vmPFC). Our findings corroborate that script-driven imagery of threat triggers neural emotional reactivity in these structures, although not all of the suggested structures showed aberrant activation. We propose therefore, particularly given the characteristics of the paradigm with regard to its potential impact on the DMN, that during script-driven imagery PD patients seem more vigilant towards possible threat, which might be mediated by the amygdala since it has been involved in the modulation of attention^96^. Attenuated activity in structures of the DMN possibly does not only mean a higher attentional focus on threat, but also a more intense struggle to contain control of threat responses, potentially mediated by the known function of the vmPFC to inhibit amygdala activation^25,43,71,89^.

Contrary to our hypotheses, the present study did not detect increased insula activation to disorder-related imagery in PD patients. This finding is surprising, since the insula is linked to interoceptive processing^97,98^ and PD patients are marked by overly sensitive attention towards interoceptive sensations^7^. In PD, hyperactivated insula was recently observed in several studies investigating emotional processing^25–27^. Therefore, we expected processing of disorder-related stimuli to evoke elevated insula activity in PD patients. Interestingly, the previous pilot fMRI study on disorder-related imagination in PD patients also did not reveal differential insula activity in PD patients relative to HC^24^. One reason for these findings might be that differential insula effects in PD patients as compared to HC are stronger in response to single highly salient threat-related stimuli^25^ and during specific emotional tasks^99^ as compared to a continuous script based symptom provocation as used in the present study. Forthcoming investigations are hence required to clarify the role of the insula during altered threat processing in PD depending on specific designs and task conditions.

Altogether, the findings of the current investigation may reflect that two opposed brain activation patterns were engaged in PD patients when exposed to disorder-related scripts: a hyperactivated pattern including the amygdala and the brainstem as well as a hypoactivated pattern including prefrontal and cingulate cortex regions. The hyperactivated amygdala-brainstem pattern may point towards hyperarousal, a hypersensitive response to potential threat, and a subsequent higher subjective level of anxiety in PD patients. The hypoactivated prefrontal/cingulate cortex pattern may reflect deficient implicit and explicit emotion suppression and increased focusing when imagining potential threat in PD patients.

We would like to mention some limitations of the present study. Although the sample size is an improvement compared to the previously published imagery fMRI study in PD^24^, a total sample size of 34 subjects (17 HC vs. 17 PD does still not admit a generalisation of the study results. Larger sample sizes would undoubtedly be beneficial not only to relate findings to interindividual differences, but also to investigate small or absent effects, such as the current brainstem effect, in more detail. Moreover, larger samples allow conducting whole-brain rather than ROI analysis, which provides stronger evidence of neural correlates of PD pathophysiology. However, several recent fMRI studies using imagery as a paradigm had comparable sample sizes^30,39,40,92^.

We also agree that medication intake poses a threat to the interpretation of study results. Thus, we exploratively excluded medicated patients from fMRI analysis, indicating that there was no confounding effect of medication intake on the present results. This finding is in line with a recent review, stating that the influence of psychotropic medication on neuroimaging findings either showed no influence, or a normalising influence^100^.

Furthermore, the present PD patient sample had comorbidities. Comorbidities represent a possible interference factor for the interpretation of the results. Patients were therefore diagnosed by an experienced clinical psychologist to ensure that PD was the principle diagnosis for all patients. Moreover, PD patients frequently show a high prevalence of comorbidities^2,101^ so that including comorbid patients might even enhance external validity. However, it would be beneficial to investigate samples of patients free of comorbidities. Finally, future studies would profit from direct comparisons between different kinds of symptom provocation designs in order to better understand the reasons between discrepancies between different studies/designs.

Concerning brainstem findings, we have to mention that we did not use high resolution imaging and specialised imaging protocols to better characterize the brain stem response, so that results have to be interpreted with some reservations.

To conclude, disorder-related relative to neutral scripts elicited increased amygdala and brainstem activity as well as decreased activity in bilateral PFC, the ACC, and the dPCC in PD patients relative to HC. Apparently, two opposed activation patterns were engaged in PD patients when exposed to threat: Firstly, imagining disorder-related scripts led to increased amygdala activity in PD patients, which might correspond to an enhanced arousal and abnormal vigilance towards threat. Elevated brainstem activation might further signify an increased awareness of bodily symptoms and a corresponding heightened subjective anxiety level during imagery of disorder-related scripts. Secondly, decreased activity in the PFC and cingulate cortex might reflect an increased attentional direction to threat and coincident deficient emotional regulation of threat. Altogether, both activation patterns might represent the neural correlates of altered threat processing during the imagination of disorder-related stimuli in PD patients.

## Methods

### Subjects

The sample consisted of 17 PD patients (male *n* = 2) and 17 HC (male *n* = 4), matched for age, gender and years of education (Table 3). All participants were right-handed and native German speakers. Participants were recruited through public advertisements and at an outpatient clinic. Patients met criteria for PD as the main diagnosis by means of the Structured Clinical Interview for DSM-IV Axis I Disorders (German version of the SCID)^102^. Furthermore, the Panic and Agoraphobia Scale (PAS) was used to confirm the diagnosis of PD^103^. This scale was used because we included PD patients with (*n* = 10) and without (*n* = 7) agoraphobia and this scale is characterised by high external validity (Pearson correlation coefficient *r* = 0.79, *p* < 0.0001 with psychiatrists’ clinical global impression of severity) and reliability (Cronbach’s *a* coefficient of 0.88; for further psychometric data see^103^). Exclusion criteria comprised neurological disorders, traumatic brain injury, psychotic or bipolar disorder, and drug abuse or dependence within the past ten years (for further sample characteristics, see Table 3). Comorbidities among PD patients comprised major depressive disorder (n = 2), somatization disorder (n = 2), dysthymia (n = 1), specific phobia (n = 1), generalised anxiety disorder (n = 2), bulimia nervosa (n = 1), obsessive-compulsive disorder (n = 1), and social anxiety disorder (n = 1). The final sample included three patients taking antidepressant medication. Informed consent has been obtained of all participants prior the experiment and the study was approved by the local ethics committee of the Medical Chamber Westphalia-Lippe and the faculty of medicine of the Westphalian Wilhelms-University of Muenster (Ethikkommission der Ärztekammer Westfalen-Lippe und der Medizinischen Fakultät der Westfälischen Wilhelms-Universität Münster). All experimental methods were performed in accordance with relevant guidelines and regulations.

### Stimuli

Stimulus material was composed of standardised disorder-related (n = 5) and neutral (n = 5) scripts. All scripts lasted exactly 30 seconds. They were recorded in third person present tense. Based on the bio-informational theory^104^, our scripts included sensory representations (i.e. stimuli conceived in the context), information about the context of the situation (who, where, what) and representations of behavioural and physiological response to the context^35^. The disorder-related scripts depicted panic-related situations (e.g. standing on a crowded platform, entering a narrow elevator) and involved suggestive descriptions of autonomic reactions (e.g. sweaty palms, palpitations). Neutral scripts described various everyday life situations (e.g. watching TV at home, reading a newspaper) and sensory information (e.g. warm water, chilly air). Scripts were tape-recorded and read out by a female voice in a neutral tone blind to the hypotheses of the study.

### Experimental procedure

Outside the scanner, participants received standardised instructions and listened to two neutral scripts via headphones during practice trials. The practice scripts were not presented during the actual experiment. Participants were encouraged to imagine the sceneries as detailed and multi-sensory as possible. If they were able to vividly imagine the scripts on the practice trials, the scanning procedure was started. In the scanner, participants were informed about the procedure by written instructions on a black screen and after practice trials via headphones. They were instructed to close their eyes during the imagination. A beep signal indicated the beginning and the end of each auditory script (400 Hz, 500 ms). The trials were pseudo-randomised with an inter-trial interval of 20 s. The participants were instructed to keep their eyes closed and to concentrate on the surrounding sounds of the scanner during the inter-trial interval. The task lasted for approximately 9 minutes. Upon finishing, participants had to evaluate the scripts on a 9-point Likert scale^105^ with regard to anxiety (1 = not anxiety-inducing, 9 = most anxiety-inducing), arousal (1 = not arousing, 9 = most arousing) and valence (1 = most unpleasant, 5 = neutral, 9 = very pleasant). Additionally, they rated their ability to imagine each script (ability to imagine the scripts, measured in %) (Table 1).

### Analysis of sociodemographic, clinical questionnaire and rating data

Sociodemographic, clinical questionnaire, and rating data were analysed using IBM SPSS software (v22, Armonk, New York, USA). Rating data were analysed using a mixed model analysis of variance (ANOVA) separately for anxiety, valence, arousal, and ability to imagine the scripts to explore within- and between-subject contrasts. A probability level of *p* ≤ 0.05 was considered as statistically significant. To resolve interaction effects, Bonferroni-corrected t-tests were implemented (corrected significance level p ≤ 0.008).

### FMRI acquisition and analysis

Data were collected using a 3 Tesla magnetic resonance scanner (“Magnetom Prisma”, Siemens, Erlangen, Germany). Before the start of the experiment, anatomical recordings of each participant were made, applying a sagittal high-resolution T1-weighted sequence with 192 slices. Functional data were collected using a T2*-weighted echo-planar sequence and comprised 260 volumes (TE = 30 ms, TR = 2080 ms, matrix = 92 × 92 voxels, field of view 208 mm^2^, flip angle 90°). Each volume consisted of 36 axial slices (thickness 3.0 mm, 0.3 mm gap, in plane resolution = 2.26 mm × 2.26 mm). The functional data were analysed using Brain Voyager QX (Version 2.8; Brain Innovation, Maastricht, The Netherlands). The first four volumes were discarded to warrant adequate steady-state tissue magnetization. The recordings were then corrected for slice time errors and motion deviation using trilinear interpolation.

Afterwards, anatomical and functional images were co-registered to fit Talairach space^106^ and smoothed spatially (6 mm full-width half-maximum [FWHM] Gaussian kernel) and temporally (high-pass filter: 10 cycles per run, low-pass filter 2.8 s; linear trend removal). The voxel-size was resampled to 2 × 2 × 2 mm for further statistical examination. Statistical analysis of *blood oxygenation level dependent (*BOLD) data consisted of multistage linear regression of the signal time course of each voxel. The signal change of each voxel in respect of the different predictors (disorder-related and neutral scripts) was modelled by a canonical hemodynamic response function (HRF) using a general linear model (GLM). Predictors of interest were the disorder-related and neutral scripts. Introducing and concluding sounds as well as the six motion parameters were considered as predictors of no interest. Z-standardised predictor estimates for the voxel-time-course were determined for each participant employing a random-effects model with adjustment for autocorrelation. Further analyses of the originated voxel-wise statistical maps were small-volume corrected for anatomically defined regions of interest (ROIs), including amygdala, insula, cingulate cortex and PFC. Based on the Automated Anatomical Labeling Atlas (AAL)^107–109^, ROIs were defined and transformed into Talairach space applying ICBM2tal^110^. For the brainstem, the ROI was downloaded from the digitised version of the Talairach atlas (http://www.talairach.org/nii/gzip/).

The ROI based voxel-level threshold was set to *p* < 0.005 to balance between Type I and II error rates (see also^111^), considering the realistic effect sizes in studies with patients^92,112^. The thresholded maps were submitted to an ROI-based correction criterion for multiple comparisons based on the estimate of the maps spatial smoothness and on an iterative procedure (Monte Carlo simulation as implemented in BrainVoyager), which do not use the Gaussian random-field approach for cluster-size thresholding. It estimated the minimum cluster-size using 10000 iterations, ensuring a false cluster-level-significance to be p < 0.05. Due to small brainstem subnuclei, no cluster threshold was used for brainstem activation^25^. Based on preceding neuroimaging studies in PD using both whole-brain^25,55^ and ROI analyses^20,24,25,113,114^, we restricted our analyses to homogeneous search spaces guided by a hypothesis-driven ROI approach. This contributes to avoid possible inflation of false-positive clusters in parametric analyses due to inhomogeneity of spatial smoothness and spatial autocorrelations across the whole brain with resulting hot spots of false positive clusters^115^. The datasets generated during and/or analysed during the current study are available from the corresponding author on reasonable request.
